# Progressive Evaluation of Ischemic Occlusion in a Macaque Monkey with Sudden Exacerbation of Infarction During Acute Stroke: A Case Report

**DOI:** 10.3390/vetsci12030231

**Published:** 2025-03-03

**Authors:** Chun-Xia Li, Xiaodong Zhang

**Affiliations:** EPC Imaging Center, Emory National Primate Research Center, Emory University, 954 Gatewood Rd NE, Atlanta, GA 30329, USA

**Keywords:** ischemic infarction, non-human primate, MRI, early neurological deterioration

## Abstract

Early neurological deterioration is associated with poor functional outcomes in stroke patients, but the underlying mechanisms remain unclear. This study aims to understand the progression of stroke-related brain damage using a rhesus monkey model with ischemic occlusion. Multiparameter MRI was used to monitor the progressive evolution of the brain lesion following stroke. Resting-state functional MRI, dynamic susceptibility contrast perfusion MRI, diffusion tensor imaging, and T1- and T2-weighted scans were acquired prior to surgery and at 4–6 h, 48 h, and 96 h following the stroke. The results revealed a sudden increase in infarction volume after the hyper-acute phase but before 48 h on diffusion-weighted imaging (DWI), with a slight extension by 96 h. Lower relative cerebral blood flow (CBF) and time to maximum (Tmax) prior to the stroke, along with a progressive decrease post-stroke, were observed when compared to other stroke monkeys in the same cohort. Functional connectivity (FC) in the ipsilesional secondary somatosensory cortex (S2) and primary motor cortex (M1) exhibited an immediate decline on Day 0 compared to baseline and followed by a slight increase on Day 2 and a further decrease on Day 4. These findings provide valuable insights into infarction progression, emphasizing the critical role of collateral circulation and its impact on early neurological deterioration during acute stroke.

## Simple Summary

Sudden deterioration of ischemic lesions during acute stroke is sometimes observed in clinical practice but is not well understood. In this study, multiparameter MRI was used to evaluate the alterations of cerebral blood flow (CBF), brain function, and brain structure in a macaque monkey at multiple time points following a stroke. A substantial increase in infarction occurred after the hyper-acute phase but before 48 h post-stroke. Key brain regions responsible for movement and sensation showed reduced functional activity. Poor collateral circulation to the affected area may contribute to the sudden worsening of brain damage during acute stroke. Also, these findings highlight the critical role of cerebral blood flow and functional connectivity measurements in understanding the evolution of ischemic lesions following stroke onset.

## 1. Introduction

Stroke is one of the leading causes of both death and long-term disability in the world [1]. In general, stroke lesions evolve progressively from the hyper-acute to the acute phase, and then the subacute phase, in which some spontaneous recovery is generally seen [2,3]. However, early neurological deterioration is sometimes seen in stroke patients and associated with poor functional outcomes, but the underlying mechanisms are still not well understood.

Rodent models have been widely used in studies of stroke disease [4,5,6]. However, nonhuman primates (NHPs) have greater translational potential than rodents, as they mimic most aspects of human, including brain anatomy, physiology, and motor and somatosensory functions, allowing comprehensive behavioral assessments [7,8,9,10,11,12,13]. NHPs with middle cerebral artery (MCA) occlusion have been established and proven to be an excellent large animal model for translational research [7,8,12,13]. In our experimental investigation of the NHP stroke model, eight animals underwent permanent MCA occlusion, and the MRI findings were reported previously [7,12,13]. The evolution of the infarct generally followed a natural logarithmic pattern during the hyper-acute phase of stroke up to 48 h post-stroke, and the temporal evolution resembled the findings seen in most stroke patients [7]. However, one animal exhibited a distinct pattern: its infarct volume remained below 1 mL during the hyper-acute phase, followed by a sudden exacerbation observed at 48 h (7.2 mL), markedly different from the infarction evolution seen in the other seven monkeys during the acute phase. Additionally, our previous study identified functional connectivity (FC) as a robust biomarker for quantifying brain function alterations in monkeys post-stroke [12]. In the present study, the longitudinal evolution of infarction in this particular monkey was examined and characterized using multiparameter MRI techniques.

## 2. Methods and Materials

An adult rhesus monkey (ID: PH1019, female, 11 kg, 13 years old) received a permanent middle cerebral artery occlusion (pMCAo) surgical procedure as reported previously [12,13,14,15]. Briefly, the subject was anesthetized with 1.0–1.5% isoflurane during the stroke surgery (1 to 1.5 h). After the occlusion of the distal M2 segment of the MCA, the animal was prepared for transport and immediately moved to the MRI suite. The animal was maintained under 1.0–1.5% isoflurane anesthesia during each MRI scan (3–6 h). Following each MRI scan before Day 4, the animal was allowed to recover from anesthesia and returned to its home cage and was under continuous 24 h video surveillance. A veterinarian conducted daily observations and assessments of the animal’s condition. The animal was provided with softened chow, along with supplemental food treats, including approved fruits, vegetables, and nuts, as well as juice, to facilitate the evaluation of swallowing difficulties and mild facial paresis. Additionally, the animal’s attitude, appetite, stool quality, overall health status, and the condition of surgical incisions were monitored and recorded daily.

### 2.1. MRI Experiments

**Figure 1:**
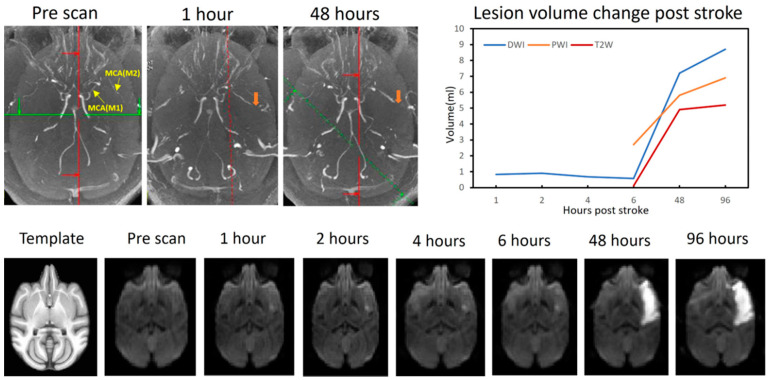
Representative MR angiography map (**top left**), lesion volume changes post-stroke (**top right**) and DWIs (**bottom**) of the monkey at different time points post-stroke. The orange arrow points to the MCA (middle cerebral artery) occlusion location. The yellow arrow points to the different branches of the MCA. DWI: diffusion-weighted images, T2W: T_2_-weighted images, PWI: perfuse-weighted images.

MR imaging was performed using a 3T clinical scanner (Siemens Medical Solutions USA, Inc., Malvern, PA, USA) and an 8-channel phased-array knee coil, with the imaging settings and protocols having been previously described [7,14,15]. The images, including MR angiography (MRA) (Figure 1), resting state functional MRI (rsfMRI), diffusion tensor images (DTI), and T1 and T2 weighted images, were obtained before surgery (pre) and at 4–6 h (Day 0), 48 h (Day 2), and 96 h (Day 4) following the stroke. Gadolinium bolus injection (0.02 mg/kg) was administrated near the end of each scan session for perfusion mapping. The rsfMRI was acquired before the Gadolinium bolus injection. Pre- and post-contrast T1-weighted structural images were obtained using the 3D MPRAGE sequence to assess potential hemorrhagic complications. The DTI protocol was repeated every hour during the Day 0 (surgical day) scan.

### 2.2. Lesion Volume Measurements

DTIs were pre-processed with FSL (https://fsl.fmrib.ox.ac.uk/fsl/docs/, accessed on 1 October 2015) at each time point. Lesion volumes were determined from diffusion-weighted images (DWI) by applying a threshold of mean + 2 × standard deviation to the DWI intensity on the contralesional side. Lesion volumes from the T2-weighted images (T2W) and perfusion-weighted images (PWI) were obtained through manual tracing. DWI infarct volumes were estimated using custom-built Matlab scripts. Lesion volumes for each measurement were estimated by summing the lesion area on each slice of the corresponding images and multiplying by the slice thickness (Figure 1) [7,12].

### 2.3. Perfusion MRI

**Figure 2:**
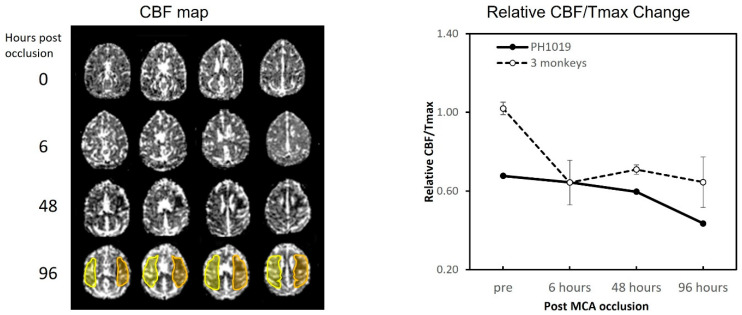
Serial axial cerebral blood flow (CBF) maps (**left**) illustrate temporal perfusion changes after permanent middle cerebral artery (MCA) occlusion and relative CBF/Tmax changes in final infarction regions of monkey brains in the monkey (PH1019) and the other 3 monkeys after MCA occlusion (**right**). The yellow and orange shaded areas represent the regions of interest (ROIs) used to measure CBF/Tmax from the contralesional (yellow) and ipsilesional (orange) areas of the brain.

Perfusion maps (CBF and Tmax) were created using the perfusion software package on a Siemens Workstation (Siemens Medical Solutions USA, Inc., Malvern, PA, USA). Regions of interest (ROIs) for the infarction area and secondary somatosensory cortex (S2) at 96 h post-stroke were delineated on the corresponding cerebral blood flow (CBF) and Tmax maps using ImageJ software (ImageJ 1.54g). The average cerebral blood flow (CBF) for Tmax ≥ 7 s was quantified for the selected ROIs in both the contralesional (CBF-Con, Tmax-Con) and ipsilesional (CBF-Ipsi, Tmax-Ipsi) brain regions (Figure 2). Relative CBF was calculated using the formula Rel-CBF = CBF-Ipsi/CBF-Con. In addition, relative Tmax was computed as Rel-Tmax = Tmax-Ipsi/Tmax-Con. The relative CBF/Tmax ratio was derived by using the formula Rel-CBF/Tmax = Rel-CBF/Rel-Tmax [12].

### 2.4. Resting-State Functional MRI

**Figure 3:**
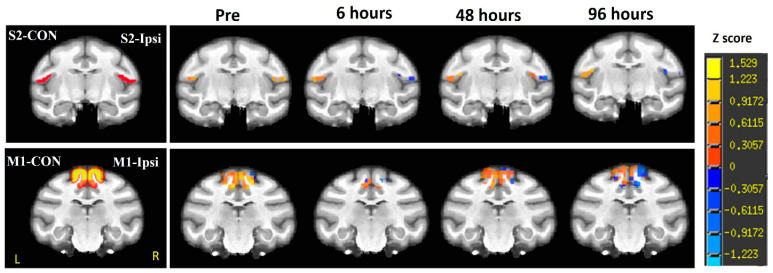
The representative template slices of the monkey brain with ROIs of contralesional S2 (S2-CON, seed for functional connectivity analysis, **top left**), contralesional M1 (M1-CON, seed used for functional connectivity analysis, **bottom left**), and ipsilateral S2 (S2-Ipsi), ipsilateral M1 (M1-Ipsi) (**left**), and representative functional connectivity maps in S2 (**top right**) and M1 (**bottom right**) before surgery (pre) and post-surgery at 6 h, 48 h, and 96 h. *p* = 0.044 with 100 voxels as threshold.

In this monkey study, each set of T1-weighted images was co-registered to the corresponding preprocessed EPI (echo planar imaging) images (i.e., after field map correction, slice timing correction, and rigid body registration) using a custom-built script of AFNI (Analysis of Functional NeuroImages). Regions of interest (ROIs) (Figure 3, left) were manually delineated on the EPI images by referencing the monkey brain atlas and corresponding T1-weight images. Time-series signals from white matter and cerebrospinal fluid in the EPI data were regressed out using a general linear model, with temporal filtering applied in the 0.009 Hz to 0.0237 Hz band-pass range and spatial smoothing via Gaussian blur (2.5 mm full width at half maximum). All procedures were implemented with an AFNI script (http://afni.nimh.nih.gov, accessed on 15 August 2024) [12].

Seed-based correlation analyses were conducted by using the average time courses extracted from the contralesional secondary somatosensory cortex (S2-CON) or contralesional primary motor cortex (M1-CON) as seed signals for generating correlation maps (Figure 3). Z-transformation was applied to convert these maps into functional connectivity (FC) maps. The FC maps for each time point—pre-surgery (pre), Day 0, Day 2, and Day 4 post-stroke—were then aligned and superimposed onto a monkey template for visualization purposes (Figure 3).

### 2.5. Neurological Examination

Spetzler neurologic deficit scoring was evaluated before surgery (baseline) and daily post-stroke, with a total score ranging from 1 to 100 (Table 1). The scoring system assigned 70 points for motor function, 5 points for visual fields, 20 points for behavior (including awareness and aggression), and 5 points for cranial nerve function with the detailed procedure reported previously [7,12,13].

### 2.6. Histology and Immunohistology

**Figure 4:**
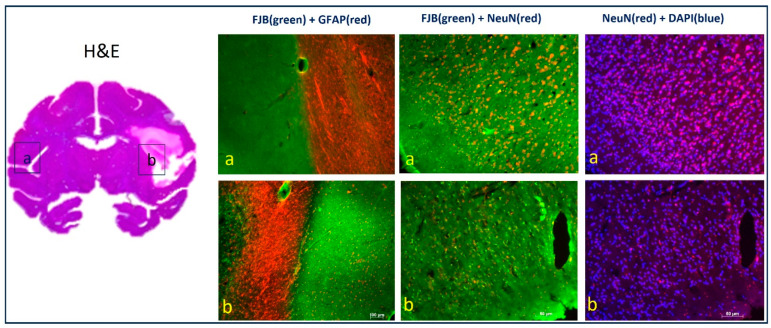
Illustration of representative brain slides of H&E (**left**), GFAP, FJB, NeuN, and DAPI staining of the monkey at 96 h post-stroke: (**a**) the contralesional S2 area, (**b**) the ipsilesional S2 area.

The brain was carefully extracted and fixed in 10% buffered formalin [7,12] after the animal was euthanized with pentobarbital (i.v., 100 mg/kg) while anesthetized. Then, the brain was blocked and sectioned into 50 µm slices using a freezing microtome. Selected sections were processed with hematoxylin and eosin (H&E) [16], and immunohistology protocols which were modified based on the previous protocols used in rodents [17], using the antibodies such as glial fibrillary acidic protein (GFAP; Cy3-conjugated anti-GFAP, 1:500, C9205, Sigma, Kanagawa, Janpan) and NeuN (neuronal nuclei; anti-NeuN 1:200, MAB314, Millipore, Billerica, MA, USA), followed by Fluoro-Jade B (FJB, AG310, Millipore, Billerica, MA, USA) or DAPI (4′,6-diamidino-2-phenylindole, D9542, Sigma) staining (Figure 4).

### 2.7. Ethical Approval Protocol

All procedures in the monkey study were approved by the Institutional Animal Care and Use Committee (IACUC, YER-2000765-030213) at Emory University. These procedures complied with the Animal Welfare Act and the Public Health Service Policy on Humane Care and Use of Laboratory Animals.

## 3. Results

### 3.1. MR Angiography and Infarction Evaluation with DWI

MCA occlusion was assessed and confirmed using MRA (Figure 1). At 1 h post-occlusion, a filling defect was observed in the M2 branch of the MCA and became more pronounced at 48 h. Ischemic infarction was seen on DWIs, primarily localized in the right frontal cortex, starting from the insular cortex and extending to S2 within 1–6 h post-occlusion (Figure 1). Subsequently, it rapidly expanded to the primary somatosensory cortex (S1), the orbital and ventral prefrontal cortex, premotor cortex, primary cortex (M1), parietal cortex, the tail of the nucleus caudate and the putamen, and even parts of the auditory cortex by 48 h post-occlusion. At 96 h, the infarction had extended to the putamen (Figure 1).

### 3.2. Lesion Evolution

As shown in Figure 1, the ischemic infarction initially localized primarily to the insula on the day of surgery (less than 1 mL until 6 h post operation). At 48 h of post-stroke, the lesion had significantly expanded to involve the S2 and M1 regions, the orbital and ventral prefrontal cortices, the premotor cortex, the parietal cortex, and even parts of the auditory cortex (~7.2 mL). This lesion expansion continued, reaching the putamen by 96 h. The lesion volume remained small and stable within the first 6 h of post-stroke; however, it then experienced a sudden increase, seen at 48 h, and continued to grow slightly thereafter, as evidenced by lesion volume measurements from DWI, PWI, and T_2_W (Figure 1, right).

### 3.3. CBF and Tmax Changes in the Lesion Regions

Temporal changes in cerebral blood flow (CBF) were assessed using perfusion MRI with gadolinium bolus injection. The evolution of perfusion over time following stroke onset is depicted in Figure 2. At 6 h post-occlusion, the abnormal CBF volume was approximately 2.7 mL, which then markedly increased by 48 h, and remained nearly stable from 48 to 96 h post-occlusion (5.8 mL vs. 6.9 mL) (Figure 1). A comparison with corresponding lesion volume changes observed in DWIs, as illustrated in Figure 1 and Figure 2, revealed a clear diffusion–perfusion mismatch at 6 h post-occlusion, which gradually decreased after 48 h.

Compared to the other three monkeys with MCA occlusion, monkey PH1019 exhibited significantly lower relative CBF/Tmax at prescan. Additionally, the low relative CBF/Tmax indicated a progressive decrease from 6 h to 96 h post-stroke, with no apparent recovery trend observed as in the other three monkeys (Figure 2, right). 

### 3.4. Functional Connectivity Changes in S2 and M1 Regions

A significant reduction in functional connectivity (FC) between the contralesional and ipsilesional S2 and M1 regions was observed immediately following the stroke on Day 0, compared to the prescan baseline; the FC transiently increased on Day 2 compared to Day 0 post-stroke (Figure 3), but subsequently decreased and was significantly lower than the prescan values on Day 4. Interestingly, the FC between the contralesional S2 and M1 regions exhibited distinct patterns. The FC within S2-CON showed no significant changes during the acute phase of stroke compared to the prescan values. In contrast, the FC within M1-CON significantly decreased on Day 0 (6 h post-stroke), recovered on Day 2 (48 h), and then continued decreasing on Day 4 (96 h post-stroke).

### 3.5. Behavior Evaluation

The animal stayed calm, alert, and responsive during each observation period. On Day 1 (24 h post-stroke), hemiparesis was noted in the left front and left hind limbs. Motor strength in the right limbs was diminished, but no complete paralysis was observed. On Days 2 and 4 (48–96 h after the stroke), the animal continued to be bright, alert, and responsive. Muscle strength in the right limbs remained weakened, and the monkey showed hesitation in moving freely within its enclosure. The neurological scores were as follows: 100 at baseline (before stroke surgery), 85 on Day 1 (24 h post-stroke), Day 2, and Day 4 (Table 1).

### 3.6. Neuropathology

Compared to the contralesional hemisphere (Figure 4a), the H&E staining results revealed ischemia-induced damage to neural and glial cells in the ipsilateral S2 regions (Figure 4b). FJB and GFAP staining demonstrated that an increase in degenerated neurons (marked by light green FJB) and glial cells (marked by red GFAP) (Figure 4b). FJB and NeuN staining showed that normal neurons (marked by red NeuN) significantly decreased, and degenerated neurons (marked by light green) obviously increased in the infarction regions. NeuN and DAPI staining showed that cell count (marked by blue DAPI) was not significantly decreased but the number of neurons (marked by red NeuN) significantly decreased in the infarction regions.

## 4. Discussion

Sudden deterioration in ischemic strokes is sometimes observed in clinical practice. In the present study, a similar case of ischemic infarction was examined in an adult macaque monkey. The lesion was induced by experimental MCA occlusion and longitudinally assessed using multiparameter MRI. The findings in diffusion, perfusion and functional MRI suggest the critical role of collateral circulation in the evolution of ischemic lesion following stroke onset.

### 4.1. MCA Stroke and MR Angiography

The MCA territory is commonly affected by cerebral infarctions in stroke patients. Branching directly from the internal carotid artery, the MCA comprises four main branches: M1, M2, M3, and M4. The frontal, temporal, and parietal lobes, along with the entire basal ganglia region, can be encompassed [18] and the motor and sensory systems are frequently affected.

In the present study, the occlusion of the distal M2 segment of the right MCA was performed and verified using c-arm fluoroscopy. The angiography around 1 h post-stroke (Figure 1) demonstrated that the M2 section was occluded and the residual section to the MCA main trunk was dilated compared to left MCA; the compensatory dilation became even more obvious at 48 h post-stroke, but the residual section was not detected at 96 h post-stroke. During the occlusion of the M2 segment, the compensatory dilation of collateral and microcirculatory vessels played a crucial role in maintaining blood supply to the MCA territory and enhancing perfusion reserve in the affected area. Such a process in microcirculatory vessels could enhance the function of brain cells in the ischemic penumbra, improve the outcome, and help safeguard the brain function from additional damage after stroke onset [19].

### 4.2. Lesion Evolution and Collateral Condition Change

As observed in Figure 1, the ischemic infarction was initially localized mainly to the insular cortex on the surgical day. At 48 h post-stroke, the lesion had significantly expanded, and the expansion of the infarction continued until 96 h. Unlike other monkeys with permanent MCA occlusion [7], the infarction volume of this monkey did not follow a logarithmic pattern during acute stroke; instead, a sudden enlargement of the infarction was observed at 48 h post-stroke. The poor collateral status likely played a significant role in the deterioration of infarction. The low relative CBF/Tmax observed in the prescan compared to the other three monkeys suggests inadequate collateral circulation in this monkey. Collateral circulation status is a key factor in determining the volume of cortical infarction [20], with robust collateral circulation generally linked to a smaller infarct core in stroke patients [21,22].

The combined CBF/Tmax ratio index has been utilized to distinguish between adequate and insufficient collateral circulation in patients with acute ischemic stroke [23] and animal models [12]. Our results indicate that the Rel-CBF/Tmax in the lesion area of the monkey was notably lower compared to those in the other three monkeys during the prescan, and it continued to decline until 96 h post-stroke, with no signs of recovery (Figure 2, right). This finding suggests that poor collateral circulation may have contributed to the rapid expansion of the lesion observed at 48 h post-stroke. The absence of a recovery trend in the ipsilesional area at 48 h post-occlusion further confirms the poor collateral circulation in this monkey (Figure 2, right). Additionally, the third piece of evidence stems from angiography. The prominent dilation of vessels observed after occlusion of the M1 segment of the MCA on the first day of stroke and 48 h post occlusion can likely be attributed to the sudden enlargement of the infarction starting from 48 h post-stroke. Compensatory dilation of collateral and microcirculatory vessels is essential in increasing blood supply to the MCA territory during infarctions.

### 4.3. Functional Connectivity Changes in S2 and M1 During Acute Stroke

Hemiparesis, characterized by weakness on one side of the body, is a common symptom of stroke in humans and animals [24,25]. In this monkey, mild hemiparesis was observed in both the left forelimb and hind limb 24 h after the stroke, and the symptom persisted until the animal was sacrificed at 96 h post-stroke. This presentation is consistent with the clinical manifestation of hemiparesis in stroke patients [24,25]. Also, a significant reduction in functional connectivity (FC) between the contralesional and ipsilesional S2 and M1 regions was observed when compared to that in the contralesional S2 and M1 regions (Figure 3) [26,27].

Our previous study identified progressive alterations in functional connectivity (FC) in the S2 of monkeys in the same cohort during acute stroke [12]. In the S2 and M1 seeded FC maps, the FC alterations (Figure 3) between the contralesional and ipsilesional hemispheres in the monkey exhibited similar patterns in the first 6 to 48 h after the stroke, in agreement with those observed in our previous FC study of the same cohort [12]. Specifically, the FC between the contralesional and ipsilesional S2 and M1 regions of the monkey decreased immediately following the stroke but showed slight recovery by 48 h [12], suggesting a rapid response in the FC of S2 and M1 during acute stroke. In contrast to the FC studies of stroke patients [28,29], our monkey results indicate that recovery effects emerge at an early stage (48 h post-stroke) in both S2 and M1 areas, suggesting that early sensory and motor compensation may facilitate the recovery process.

The sustained increase in functional connectivity (FC) between S2-CON and S2-ipsi was observed from Day 2 to Day 4 post-stroke in the other monkeys of the same cohort [12], but not in this monkey. The relative CBF/Tmax results suggested that this monkey did not show robust collateral circulation, as seen in the S2 area of the other three monkeys (Figure 3). Accordingly, the temporary increase in FC observed in the S2 and M1 regions of the monkey at 48 h post-occlusion may not be sustained. This finding may help explain why this monkey did not exhibit a trend toward neurological recovery at 96 h post-stroke, in contrast to the other three monkeys [12].

It is worth noting that the functional connectivity (FC) within the contralesional S2 and M1 regions of this monkey exhibited distinct evolutionary patterns. The FC within S2-CON showed no significant changes during the acute phase of stroke compared to pre-stroke values, consistent with the other three monkeys [12]. However, the FC within M1-CON significantly decreased on Day 0 (6 h post-stroke), recovered on Day 2 (48 h), and then continued to decrease on Day 4 (96 h). These changes suggest that contralesional M1 function could be influenced early in the stroke evolution of this monkey. The diminished motor strength in the right limb on Day 1 may reflect dysfunction in the contralesional M1 on Day 0 post-stroke.

In early-stage stroke patients, previous studies have identified a relationship between abnormal contralesional M1 function and motor performance [28]. Additionally, research has shown that severely impaired stroke patients often exhibit an early pattern of upregulated contralesional motor network connectivity [29]. Therefore, the observed dysfunction in contralesional M1 in this study may signal the sudden exacerbation of infarction on Day 2 post-stroke. Therefore, FC in contralesional M1 may provide important, even predictive insights into prognosis and the recovery process following stroke [30].

## 5. Conclusions

The present study provides valuable insights into the sudden exacerbation and progression of infarction following stroke onset, highlighting the crucial role of collateral circulation in the compensatory mechanisms of stroke brains. The findings also emphasize the role of functional connectivity and blood flow measurements in examining early, abrupt neurological deterioration of the subject during acute stroke.

## Figures and Tables

**Table 1:** Spetzler neurologic evaluation points and scores of the monkey. Post-op, post operation.

Test Items	Points	Baseline	Baseline	Baseline	24 Hours Post-op	42 Hours Post-op	72 Hours Post-op	96 Hours Post-op
**Motor Function**								
Severe hemiparesis	10							
Mild hemiparesis	25							
Normal strength: Favors opposite extremity	55				55	55	55	55
Normal strength: Normal function	70	70	70	70				
**Behavior**								
Death	0							
Coma	1							
Aware of surroundings: Not active	5							
Aware of surroundings: Moves in response to examiner	15							
Normal aggression	20	20	20	20	20	20	20	20
**Ocular and cranial nerve function**								
Facial movement: Paretic	1							
Facial movement: Normal	5	5	5	5	5	5	5	5
Visual Field: Hemianoptic	1							
Visual Field: Normal	5	5	5	5	5	5	5	5
**Total score**	100	100	100	100	85	85	85	85

## Data Availability

The datasets produced and examined during this study can be accessed from the corresponding author upon reasonable request.
